# Stressed, sick, and sad: Neuroendoimmune pathways between subjective lifetime stress and depression

**DOI:** 10.1016/j.bbih.2021.100249

**Published:** 2021-03-31

**Authors:** Katherine Gardhouse, Dean Carcone, Anthony C. Ruocco

**Affiliations:** Department of Psychology, University of Toronto Scarborough, 1265 Military Trail, Toronto, Ontario, M1C 1A4, Canada

**Keywords:** Cortisol, Depression, Inflammation, IL-6, Neuroendoimmune system, Stress

## Abstract

Disruptions in stress-sensitive biological systems, notably the immune system and hypothalamic-pituitary-adrenal axis, are strongly implicated in depression, and disturbances in these neuroendoimmune systems could reflect potential pathways through which experiences of stress are translated into depression. To characterize the links between stress and depression, the present study investigated whether neuroendoimmune activity mediates the relationship between perceived stress and depressive symptoms in 59 medically healthy adult females with varying levels of depression. Consistent with hypotheses, both greater perceived stress and higher concentrations of the proinflammatory immune marker, interleukin-6 (IL-6), were associated with greater depressive symptoms. Although neuroendoimmune activity did not significantly mediate the relationship between lifetime perceived stress and depressive symptoms, when considered together, elevated concentrations of IL-6 and lower free cortisol mediated the relationship between severity of childhood stress and current depressive symptoms. These findings shed light on how early life stress may be translated into adulthood depression.

## 1 Introduction

Neuroendoimmunological research has produced evidence of a dynamic interconnection between stress and biological responses from the immune system and hypothalamic-pituitary-adrenal (HPA) axis (Ménard et al., 2017). Dysregulation (i.e., both increases and decreases in activity) of these systems in isolation is reported extensively in relation to depression and may directly contribute to depressive symptoms (Maes et al., 2012; Ménard et al., 2017). However, capturing the interplay of these systems in relation to both stress and depression has not been comprehensively addressed.

Evidence from a mixed-gender sample suggests that an exaggerated cortisol response under medically induced inflammatory challenge is associated with a higher risk of developing a major depressive episode (Capuron et al., 2003). However, sex-specific investigations demonstrate the reverse in females, with hypocortisolism in the presence of immune activity associated with depressive symptom severity in one study (Suarez et al., 2015), and an acute laboratory stress paradigm in another (Miller et al., 2005). Given that immune-related illnesses are more prevalent in females (Desai and Brinton, 2019), and that females are 1.5–3 times more likely to experience depression than males (American Psychiatric Association, 2013), understanding sex-specific neuroendoimmune disruptions may provide key insights into depression vulnerability.

Beyond well-known variability in neuroendocrine responses across the sexes, the timing and severity of stressors may play a critical role in neuroendoimmune disruptions. Similar patterns of hypocortisolism have been found in many chronic stress populations, such as posttraumatic stress disorder (PTSD), caregivers of ill family members, irritable bowel syndrome, chronic fatigue, and fibromyalgia (e.g., Bauer et al., 2010). In a meta-analysis of chronic stressors with 8521 participants, an inverse relationship was found between onset of chronic stress and daily cortisol output, such that recent traumas are associated with higher cortisol measurements across the day, and more distant traumas are associated with hypocortisolism in the morning and flattened diurnal slopes (Miller et al., 2007). Given that cortisol plays a critical role in regulating the magnitude and duration of immune activity, and, in turn, immune activity may directly contribute to depressive behaviours (Maes et al., 2012, Ménard et al., 2017), analyzing cortisol directly in relation to immune activity may provide a valuable approach to capture underlying neuroendoimmune disruptions that increase one’s vulnerability to depression (Suarez et al., 2015).

To examine the relationship of specific time periods of life stress with depression, we utilized a cross-sectional approach to investigate the extent to which neuroendoimmune activity (levels of both free cortisol and immune activity) mediates the relationship between stress experienced across the lifespan and depression severity in adulthood. We hypothesized that neuroendoimmune activity would mediate the relationship between cumulative life stress severity (i.e., the severity of subjective ratings of stress experienced across the lifespan) and depression. Exploratory analyses were planned to examine this relationship within the context of childhood stress severity (i.e. severity ratings of stressful experiences prior to age 13).

## 2 Methods

### 2.1 Participants

Females with varying levels of depression were recruited to investigate dimensional associations of depressive symptoms with stress and neuroendoimmune biomarkers, rather than drawing comparisons between groups with clinical diagnoses. To ascertain participants with a range of depressive symptoms, three groups were recruited to achieve adequate sampling of depression symptoms and cumulative lifetime stress across the full spectrum of severity: (a) participants diagnosed with a depressive disorder (major depressive disorder [MDD] and/or persistent depressive disorder [PDD]) and borderline personality disorder (BPD), who were expected to have the most severe and persistent depressive symptoms and exposure to stress; (b) participants with a depressive disorder but without BPD, who were anticipated to have a mild-to-moderate severity of depressive symptoms and stress exposure; and (c) participants without a depressive disorder or BPD (although other psychiatric diagnoses were permitted), to capture subthreshold levels of depressive symptoms, as well as the absence of depressive symptoms, and the lowest levels of stress exposure. Eligibility criteria included medically healthy, female adults (ages 18–55) who were English-speaking and right-handed (for inclusion in a larger fMRI study). Exclusion criteria for all participants included a lifetime diagnosis of a psychotic disorder, bipolar disorder, current eating disorder, serious medical or neurological illness, neurodevelopmental disorder, moderate or severe alcohol or substance use disorder within the past three months, pregnancy, lactation, or current antibiotic or anti-inflammatory drug use.

Participants provided written informed consent and completed a fasted (minimum of 8 ​h) blood draw between 8 a.m. and 9 a.m. on the day of testing. Prior to providing a blood sample, participants agreed to abstain from drug and alcohol use for two days before testing. They were instructed to avoid anti-inflammatory medications and intense physical exercise for 24 ​h prior to study participation and to aim for at least 8 ​h of sleep the night before participating in the study.

### 2.2 Clinical measures

The Structured Interview for DSM-5 was used to determine the presence of psychiatric diagnoses relevant to the study’s eligibility criteria. The interview was administered by trained Ph.D. students and supervised by a licensed psychologist. The 17-item Hamilton Depression Rating Scale (HAMD; Hamilton, 1960) and the Beck Depression Inventory-II (BDI-II; Beck et al., 1996) were used to assess depression severity. Scores from the BDI-II were used as the primary outcome variable in the analyses. The Stress and Adversity Inventory for Adults (STRAIN), is a computerized measure with good psychometric properties that was self-administered to characterize lifetime experiences of stress (Slavich and Shields, 2018).

### 2.3 Biomarkers

Biomarkers of stress were measured via free cortisol and proinflammatory cytokine assays. IL-6 was selected as the primary proinflammatory immune marker in this study because of its consistent relationship with both stress and depression (Osimo et al., 2020). However, additional proinflammatory immune markers (IL-1β, tumor necrosis factor-alpha [TNF-α], C-reactive protein [CRP]) were assayed for exploratory analyses based on prior research implicating them in stress and depression (Haapakoski et al., 2015; Osimo et al., 2020). Plasma was separated through centrifugation and transferred into 500 ​μL aliquots and stored at −80 ​°C until analysis. IL-6, IL-1β, and TNF-α were measured by Bioplex 200 multiplex immunoassay system (BioRad, USA). The 4-plex plate was included in a kit from BioRad including all standards and reagents. The analysis was carried out following established manufacturer protocols. The quantification range for these assays are 0.27–4457 ​pg/mL for IL-1β, 0.4–6557 ​pg/mL for IL-6, and 3.16–51852 ​pg/mL for TNF-α, respectively. Analysis of high sensitivity CRP (hsCRP) was conducted with a routine certified clinical assay for cardiovascular risk assessment. Free cortisol assay was conducted using a liquid chromatography-tandem mass spectrometry (LC-MS/MS) methodology (Huang et al., 2007). Linear range was from 5nmol/L to 500 ​nmol/L.

### 2.4 Statistics

The statistical package lavaan in R was used to estimate structural equation modeling. Bootstrapping with 10,000 resamples was used to estimate standard errors and indirect effects for all mediation analyses to make analyses more robust to potential violations of the normality assumption. Additionally, 95% confidence intervals (*CI*) were computed to quantify the margin of error around effects. Although medication use, body mass index (BMI), and menstrual cycle were included as covariates in the primary analysis between IL-6 and depressive symptom severity, the *a priori* decision not to include covariates in the mediation models was made based on power estimates.

## 3 Results

### 3.1 Preliminary analyses

In total, 64 participants consented to participate in the study (see Supplementary Material for descriptive statistics). At the time of the psychodiagnostic assessment, 28 participants met criteria for a full episode of MDD (nine of whom also met criteria for PDD), 10 participants were determined to be in partial remission from a major depressive episode (one whom also met criteria for PDD), two participants met criteria for PDD with no current major depressive episode, and 22 participants did not meet criteria for any current depressive episode. According to severity ratings assessed by the HAMD, 31.7% of participants reported depressive symptoms in the “normal” range, 20.6% in the “mild-moderate” range, and 47.6% in the “severe” range. Prior to completing any testing procedures, one participant discontinued participation as they were deemed MRI incompatible and was thus excluded from participating in the study. Following the completion of data collection, three participants were removed from the final dataset due to moderate to severe substance use disorder identified during the structured clinical interview, and one due to an incidental MRI finding following neurological consult. After study exclusions, 59 individuals remained in the final sample for analysis.

After biomarker extraction, 29 participants produced IL-6 levels below the range of detection, three had TNFα below the limits of detection, and one participant’s plasma sample had insufficient supply to complete the morning free cortisol assay. Of note, assays for IL-1β were also conducted but were below the detectable limit in this sample. Limited detection of IL-1β and null associations have been typical in other MDD samples (Haapakoski et al., 2015; Osimo et al., 2020). All immune marker data were positively skewed (corrected via bootstrapping) (see Table 1 for the correlation matrix).

**Table 1:** Means, standard deviations, and bivariate correlations.

Variable	*M*	*SD*	1	2	3	4	5	6	7
1. BDI-II	25.2	17.0	1	.44∗	.43∗∗∗	.57∗∗	-.42	.47∗∗	.25
2. Cumulative Life Stress	66.6	35.4		1	.58∗∗∗	.16	-.47	.23	.03
3. Childhood Stress	20.6	15.3			1	.36	-.38	.36	.25
4. IL-6	1.1	1.0				1	-.12	.53∗	.61∗∗
5. Free Cortisol	21.5	8.5					1	.05	.13
6. TNFα	10.3	7.0						1	.32
7. CRP	3.0	4.3							1

*Note n ​=* 59 with the exception of biomarkers values. Biomarkers with quantification ranges below the limits of detection were removed from the primary analysis. After removal, IL-6 had an *n* ​= ​30, free cortisol *n ​=* 58, and TNFα *n* ​= ​56. ∗*p* ​< ​.05. ∗∗*p* ​< ​.01. ∗∗∗*p* ​< ​.001.

### 3.2 Cumulative lifetime stress, neuroendoimmune activity, and depression

Cumulative lifetime stress severity (*b* ​= ​0.46, *p* = <.01), and IL-6 (*b* ​= ​10.78, *p* = <.01) were each significantly associated with depressive symptom severity. A one-point increase in cumulative life stress severity as measured by the STRAIN was associated with a 0.46-point increase in BDI-II scores, *CI* (0.22, 0.71). Similarly, a 1pg/mL increase in IL-6 concentration in the blood plasma is associated with a 10.78-point increase in BDI-II scores, *CI* (4.62, 16.95). When covariates of medication use (including both psychopharmaceuticals and birth control), BMI, and menstrual cycle were analyzed, the relationship between IL-6 and BDI-II scores remained significant, *b* ​= ​7.90, *p* = <.01, *CI* (2.74, 13.05), suggesting that the covariates did not account for a significant portion of the variance in depression scores explained by IL-6.

**Fig. 1:**
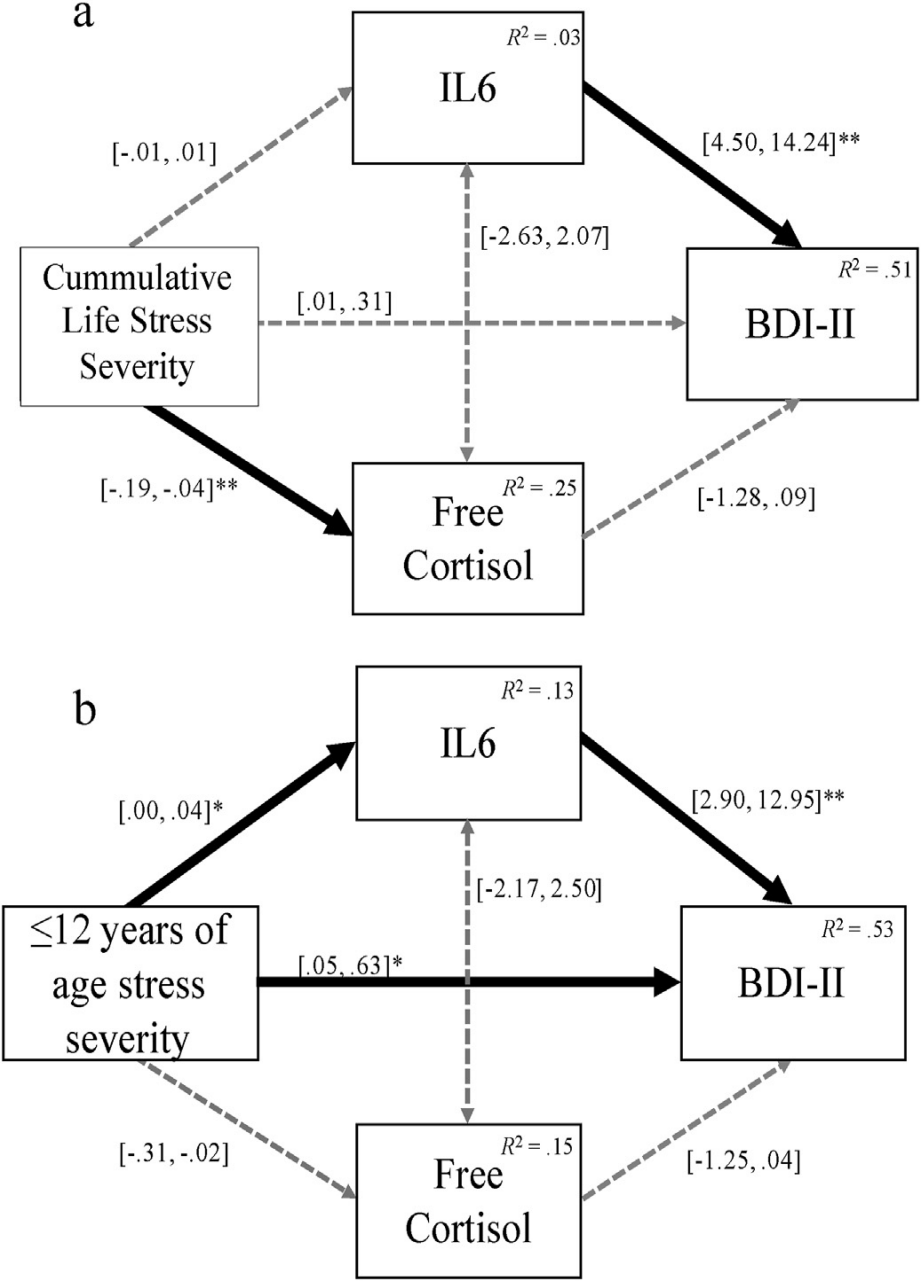
Results of Primary and Exploratory Mediation Analyses. Parallel mediation was used to test whether neuroendoimmune activity (IL-6 and free cortisol) mediates the relationship between appraisals of cumulative life stress (Fig. 1a) and childhood stress (≤12 years of age; Fig. 1b) as measure by the STRAIN and depressive symptom severity measured by the BDI-II. Bracketed numbers represent 95% confidence intervals; solid lines indicate significant regression pathways; dotted grey lines indicate relationships that were not significant.

To examine whether neuroendoimmune activity mediates the relationship between cumulative lifetime stress and depressive symptom severity, stress was entered as the independent variable, IL-6 and free cortisol as the mediators, and depressive symptom severity as the dependent variable in a parallel mediation model (see Fig. 1(a)). Examining the total effect of the parallel mediation model, cumulative lifetime stress severity was significantly associated with depressive symptom severity, *b* ​= ​0.26, *p* = <.01. Although the combined indirect effect did influence the outcome by reducing the impact of cumulative life stress on depression severity, the combined indirect effect of the mediators did not reach significance. As such, no mediation effect was detected between cumulative life stress and depression severity.

### 3.3 Exploratory analyses

The relationships of other immune markers (TNFα and CRP) with stress and depression are presented in Table 1. Although both TNFα, *r* ​= ​0.53, *p* = <.05, and CRP, *r* ​= ​.61, *p* = <.001, shared a positive relationship with IL-6, they were not significantly correlated with other markers of stress or depressive symptom severity. Given these null findings, no exploratory analyses were conducted with these additional immune markers.

An exploratory mediation analysis applying the same parallel mediation model as above was conducted to determine whether neuroendoimmune activity mediates the relationship between childhood stress and depression (see Fig. 1(b)). Examining first the total effect of the model, ratings of childhood stress were significantly associated with depressive symptom severity, *b* ​= ​0.59, *p* = <.01, *CI* (0.29, 0.88). The combined effect of the mediators significantly decreased the total effect of the model, *b* ​= ​0.25, *p* = <.05, suggesting that collectively, there is a mediation effect when both mediators are included. Here, when there is a 1pg/mL increase in IL-6 *and* a 1nmol/L decrease in free cortisol, there is a 0.25-point increase in BDI-II scores. When both mediators were added to the model, the total effect was reduced by 42.3%. As such, it can be concluded that neuroendoimmune activity mediated the relationship between childhood stress appraisals and depressive severity in adulthood.

## 4 Discussion

We utilized a cross-sectional research design to investigate the associations among cumulative lifetime stress, IL-6 activity, and depressive severity. Further, we examined whether neuroendoimmune activity mediates the relationship between childhood stress and depression severity in adulthood. We found links among cumulative lifetime stress, neuroendoimmune activity, and depression. On average, individuals with higher IL-6 reported greater depressive symptoms. In total, IL-6 accounted for 32% of the variance in depressive symptoms in the sample. This finding is consistent with other similar studies, including several meta-analyses (e.g., Haapakoski et al., 2015; Osimo et al., 2020).

In exploratory analyses, we determined that immune and HPA axis biomarkers were associated with depression and mediated the relationship between childhood stress and current adulthood depression severity. Specifically, elevations in IL-6 and lower morning levels of free cortisol contributed to the mediation effect between childhood stress appraisals and adulthood depressive severity. This pattern of high immune activity and low cortisol is theoretically consistent in relation to more severe levels of depression and stress (Ménard et al., 2017) and has been documented in relation to depression in female samples (Suarez et al., 2015; Miller et al., 2005).

Childhood stress and trauma are potent risk factors for depression in adulthood (Danese and Baldwin, 2017). The possibility that neuroendoimmune disruptions may be one mechanism through which these vulnerabilities are sustained has significant implications. Such findings may signal the influence of these stress-sensitive biological systems, especially during critical periods of development, on vulnerability to depression across the lifespan (Danese and Baldwin, 2017). It is important to consider, however, that the mediation analyses carried out in the present study were based on cross-sectional data. Therefore, the results reflect observed associations and do not infer or prove causality. However, the components of the models and the model parameters were selected based on theoretical rationale and prior research demonstrating a temporal sequencing of the variables (e.g., that immune activity most commonly precedes depressive symptoms; Huang et al., 2019). Similarly, all metrics of stress utilized in the present study incorporated time periods of stress appraisals (i.e., cumulative lifetime, early childhood) that either precede and/or subsume the period of depression being analyzed (i.e., past two weeks) to strengthen the assumptions of the mediation models. To uncover the potential causal effects of childhood stress on stress-sensitive biological systems and the downstream impacts on depression in adulthood, prospective longitudinal research is needed.

Biological sex has a significant moderating effect on stress biomarkers with some studies reporting higher ACTH and cortisol response and less reliable elevations in proinflammatory cytokines under chronic stress conditions in males compared to females (Birur et al., 2017). Given that the bulk of immune-depression research consists of mixed-gender samples that are unbalanced and underpowered to examine sex-specific patterns (Haapakoski et al., 2015; Osimo et al., 2020), future studies may prefer to opt for sex-specific investigations to prevent confounding results.

In the present research, a significant portion of immune markers were below the limits of detection. The decision to exclude these individuals from the relevant analyses was done to provide a more conservative analysis, although some researchers choose to assign values at the lower limits of detection (Maes et al., 2012). Unobserved immune concentrations are commonly reported in the literature (Haapakoski et al., 2015; Osimo et al., 2020). To prevent these losses, future research may prefer to select methodological approaches more appropriately suited to molecules at these low quantities in both non-psychiatric and depressed samples or adopt functional genomic techniques that examine immune response genes to provide a more fine-grained transcriptional profile of immune activation (Almeida and Turecki, 2017).

The findings of our study were identified in a transdiagnostic sample of participants who carried diagnoses of many stress-linked disorders (e.g., MDD, BPD, PDD, and PTSD). This signals the possibility that there are common neuroendoimmune pathways that may lead to these biobehavioral relationships regardless of the specific psychiatric diagnosis, although more research is needed to confirm and extend these initial findings. Research adopting both transdiagnostic and dimensional approaches to study the relationships among stress, neuroendoimmune activity, and depression is crucial for advancing understanding of the neurobiology of depression and is consistent with neuroscience-based research frameworks for psychiatric illness (e.g., NIMH Research Domain Criteria initiative). Overall, this study provides new insights into potential pathways among stress, the neuroendoimmune system, and depression, shedding light on how early life stress may be translated into depression later in life.

## References

[bib1] Almeida D., Turecki G. (2017). Recent progress in functional genomic studies of depression and suicide. Current Genetic Med. Rep..

[bib19] American Psychiatric Association (2013). Diagnostic and Statistical Manual of Mental Disorders 4th Edition TR.

[bib2] Bauer M.E., Wieck A., Lopes R.P., Teixeira A.L., Grassi-Oliveira R. (2010). NeuroImmunoModulation.

[bib3] Beck A., Steer R., Brown G. (1996).

[bib4] Birur B., Amrock E.M., Shelton R.C., Li L. (2017). Sex differences in the peripheral immune system in patients with depression. Front. Psychiatr..

[bib5] Capuron L., Raison C.L., Musselman D.L., Lawson D.H., Nemeroff C.B., Miller A.H. (2003). Association of exaggerated HPA axis response to the initial injection of interferon-alpha with development of depression during interferon-alpha therapy. Am. J. Psychiatr..

[bib6] Danese A., Baldwin J.R. (2017). SSRN.

[bib7] Desai M.K., Brinton R.D. (2019). Autoimmune disease in women: endocrine transition and risk across the lifespan. Front. Endocrinol..

[bib8] Haapakoski R., Mathieu J., Ebmeier K.P., Alenius H., Kivimäki M. (2015). Cumulative meta-analysis of interleukins 6 and 1β, tumour necrosis factor α and C-reactive protein in patients with major depressive disorder. Brain Behav. Immun..

[bib9] Hamilton M. (1960). A rating scale for depression. J. Neurol. Neurosurg. Psychiatr..

[bib10] Huang M., Su S., Goldberg J., Miller A.H., Levantsevych O.M., Shallenberger L., Vaccarino V. (2019). Longitudinal association of inflammation with depressive symptoms: a 7-year cross-lagged twin difference study. Brain Behav. Immun..

[bib11] Huang W., Kalhorn T.F., Baillie M., Shen D.D., Thummel K.E. (2007). Determination of free and total cortisol in plasma and urine by liquid chromatography-tandem mass spectrometry. Ther. Drug Monit..

[bib12] Maes M., Mihaylova I., Kubera M., Ringel K. (2012). Activation of cell-mediated immunity in depression: association with inflammation, melancholia, clinical staging and the fatigue and somatic symptom cluster of depression. Prog. Neuro Psychopharmacol. Biol. Psychiatr..

[bib13] Ménard C., Pfau M.L., Hodes G.E., Russo S.J. (2017). Immune and neuroendocrine mechanisms of stress vulnerability and resilience. Neuropsychopharmacology.

[bib14] Miller G.E., Chen E., Zhou E.S. (2007). If it goes up, must it come down? Chronic stress and the hypothalamic-pituitary-adrenocortical axis in humans. Psychol. Bull..

[bib15] Miller G.E., Rohleder N., Stetler C., Kirschbaum C. (2005). Clinical depression and regulation of the inflammatory response during acute stress. Psychosom. Med..

[bib16] Osimo E.F., Pillinger T., Rodriguez I.M., Khandaker G.M., Pariante C.M., Howes O.D. (2020). Inflammatory markers in depression: a meta-analysis of mean differences and variability in 5,166 patients and 5,083 controls. Brain Behav. Immun..

[bib17] Slavich G.M., Shields G.S. (2018). Assessing lifetime stress exposure using the stress and adversity inventory for adults (adult STRAIN): an overview and initial validation. Psychosom. Med..

[bib18] Suarez E.C., Sundy J.S., Erkanli A. (2015). Depressogenic vulnerability and gender-specific patterns of neuro-immune dysregulation: what the ratio of cortisol to C-reactive protein can tell us about loss of normal regulatory control. Brain Behav. Immun..

