# Supplementary material for: Stressed, sick, and sad: Neuroendoimmune pathways between subjective lifetime stress and depression
Source: Brain Behav Immun Health. 2021 Mar 31;14:100249. doi: 10.1016/j.bbih.2021.100249 (PMC8474676; doi:10.1016/j.bbih.2021.100249)
Supplement: Multimedia component 1 [file mmc1.docx]

**Supplementary Material**

| Characteristic | Quantity and standard deviation |
| --- | --- |
| Age | 28.7 (9.1) |
| Sex (female) | 100% |
| Education Levels |  |
| High School or GED | 12.7% |
| College or University | 61.9% |
| Graduate/Professional school  <High School | 23.8%  1.6% |
| Ethnicity |  |
| Caucasian | 53.9% |
| Chinese | 11.1% |
| South Asian | 6.3% |
| Black | 3.2% |
| Southeast Asian | 3.2% |
| West Indian | 3.2% |
| Japanese | 1.6% |
| Korean | 1.6% |
| Mixed race | 12.6% |
| Other | 3.2% |
| Income |  |
| <$5000  $5000-$10,000 | 3.2%  1.6% |
| $10,000-$15,000 | 3.2% |
| $15,000-$20,000  $20,000-$25,000  $25,000-$35,000  $35,000-$50,000  $50,000-$75,000  $75,000-$100,000  $100,000-$150,000  $150,000-$200,000 | 4.8%  3.2%  9.5%  23.8%  3.2%  12.7%  19.0%  4.8% |
| >200,000 | 3.2% |
| Employment |  |
| Full-time employment | 33.3% |
| Part-time employment | 30.2% |
| Unemployed | 9.5% |
| Student  Disabled | 33.3%  6.3% |
| Homemaker | 1.6% |
| BMI |  |
| Underweight (<18.5) | 1.6% |
| Normal (18.5 – 24.9) | 55.6% |
| Overweight (25.0 – 29.9) | 27.0% |
| Obese (>30.0) | 15.8% |

*Supplementary Table 1.* *Participant Demographic Information Collapsed Across Recruitment Groups (n* = 63).

| Characteristic | Quantity and standard deviation |
| --- | --- |
| Current MDD | 44.4% |
| 2 previous episodes of MDD | 17.5% |
| ≥3 previous episodes of MDD | 27.0% |
| Mean number of MDD episodes | 3.7 (6.9) |
| PDD | 19.0% |
| HAMD Scores |  |
| Normal (≤7) | 31.7% |
| Mild (8 – 16) | 11.1% |
| Moderate (17 – 23) | 9.5% |
| Severe (≥24) | 47.6% |
| BPD | 31.7% |
| PTSD | 15.9% |
| Current alcohol use disorder | 6.3% |
| Past alcohol use disorder | 12.7% |
| Current substance use disorder | 1.6% |
| Past substance use disorder | 17.5% |
| Current nicotine/tobacco use |  |
| Daily cigarette use | 3.2% |
| Daily vape use | 1.6% |
| Age of first contact with mental health services | 18.3 (8.2) |
| Past hospitalization | 33.3% |
| Suicide attempts | 31.7% |
| Reported family history of mental illness | 61.9% |
| Previous or current psychotherapy | 71.4% |
| Currently taking psychoactive medication | 50.8% |
| Antidepressant | 46.0% |
| Antipsychotic | 15.9% |
| Tranquilizer | 11.1% |
| Stimulant | 7.9% |
| Anticonvulsant | 4.8% |
| Antiparkinsonian | 3.2% |
| Sedative | 3.2% |
| Cannabinoid (prescribed) | 3.2% |
| Opioid antagonist | 1.6% |
| Opioid (prescribed) | 1.6% |
| Contraceptives | 33.3% |

*Supplementary Table 2.* *Diagnostic and Clinical Characteristics Collapsed Across Recruitment Groups (n* = 63). HAMD = Hamilton Depression Rating Scale; MDD = major depressive disorder; PDD = persistent depressive disorder; OCD = obsessive compulsive disorder; BPD = borderline personality disorder; PTSD = posttraumatic stress disorder.

| Marker | Controls | MDD | MDD+BPD |
| --- | --- | --- | --- |
| IL-6 (pg/mL) | .85 | 1.09 | 1.28 |
| Free Cortisol (nmol/L) | 23.79 | 20.11 | 20.51 |
| TNFα (pg/mL) | 10.19 | 9.69 | 10.92 |
| CRP (mg/L) | 3.38 | 2.17 | 3.52 |

*Supplementary Table 3. Mean Concentrations for Biomarkers Across Recruitment Groups.*

Biomarkers with quantification ranges below the limits of detection were removed from the primary analysis. After removal, IL-6 had an *n* = 30, free cortisol *n =* 58*,* and TNFα *n* = 56*.* **p* < .05. ***p* < .01. ****p* < .001. IL, interleukin; TNF-α, tumor necrosis factor-α; CRP, C-reactive protein; mg/L: milligrams/Litre; pg/mL: picograms/millilitre; nmol/L: nanomoles/Litre.

| Characteristic | Controls | MDD | MDD+BPD |
| --- | --- | --- | --- |
| Age | 27.3 (8.1) | 30.0 (9.3) | 28.8 (9.8) |
| Education Levels |  |  |  |
| High School or GED | 10% | 13.0% | 20% |
| College or University | 60% | 60.8% | 60% |
| Graduate/Professional school  <High School | 30% | 21.7%  4.3% | 20% |
| Ethnicity* |  |  |  |
| Caucasian | 20.0% | 47.8% | 85.0% |
| Chinese | 10.0% | 17.4% |  |
| South Asian | 15.0% | 4.3% |  |
| Black | 20.0% |  |  |
| Southeast Asian | 5.0% |  | 5.0% |
| West Indian |  | 4.3% | 5.0% |
| Japanese |  | 4.3% |  |
| Korean |  | 4.3% |  |
| Mixed race | 25.0% | 13.0% |  |
| Other | 5.0% | 4.6% | 5.0% |
| Income |  |  |  |
| <$5000  $5000-$10,000 |  |  | 5.0% |
| $10,000-$15,000 | 5.0% | 5.0% |  |
| $15,000-$20,000  $20,000-$25,000  $25,000-$35,000  $35,000-$50,000  $50,000-$75,000  $75,000-$100,000  $100,000-$150,000  $150,000-$200,000 | 10.0%  30.0%  5.0%  15.0%  25.0% | 10.0%  30.0%  5.0%  15.0%  25.0% | 5.0%  10.0%  5.0%  25.0%  15.0%  20.0%  5.0% |
| >200,000 | 10.0% | 10.0% |  |
| Employment* |  |  |  |
| Full-time employment | 20.0% | 39.1% | 40.0% |
| Part-time employment | 40.0% | 26.1% | 25.0% |
| Unemployed | 15.0% | 4.3% | 10.0% |
| Student  Disabled | 45.0% | 26.1%  13.0% | 25.0%  15.0% |
| Homemaker | 5.0% |  |  |
| BMI |  |  |  |
| Underweight (<18.5) | 5.0% | 4.4% | 10.0% |
| Normal (18.5 – 24.9) | 55.0% | 56.6% | 40.0% |
| Overweight (25.0 – 29.9) | 30.0% | 26.0% | 25.0% |
| Obese (>30.0) | 10.0% | 13.0% | 25.0% |

*Supplementary Table 4. Participant Demographic Information Across Recruitment Groups n* = 22 controls; 23 MDD; 18 MDD+BPD; *Note.* Groups were not found to statistically differ on variables of age, education, income, or BMI.

| Characteristic | Controls | MDD | MDD+BPD |
| --- | --- | --- | --- |
| Major depressive episode (MDE) |  | 91.3% | 95.0% |
| 2 previous MDEs |  | 23.8% | 20% |
| ≥3 previous MDEs | 4.5% | 66.6% | 65.0% |
| Mean number of MDEs | 0.4(1.1) | 6.6 (10.2) | 4.2 (4.5) |
| PDD |  | 30.4% | 33.3% |
| HAMD Scores |  |  |  |
| Normal (≤7) | 86.3% | 4.7% |  |
| Mild (8 – 16) | 9.1% | 14.4% | 10.0% |
| Moderate (17 – 23) |  | 9.5% | 20.0% |
| Severe (≥24) | 4.5 | 71.4% | 70.0% |
| BPD |  |  | 100.0% |
| PTSD |  | 17.4% | 27.8% |
| Current alcohol use disorder | 4.5% | 9.0% | 5.0% |
| Past alcohol use disorder | 4.5% |  | 35.0% |
| Current substance use disorder |  | 4.7% |  |
| Past substance use disorder | 4.5% | 4.7% | 45.0% |
| Current nicotine/tobacco use |  |  |  |
| Daily cigarette use |  |  | 10.0% |
| Daily vape use |  |  | 10.0% |
| Age of first contact with mental health services | 22.5 (6.5) | 19.6 (8.8) | 16.5 (6.1) |
| Past hospitalization | 4.5% | 38.1% | 55.0% |
| Suicide attempts | 0.04 (0.2) | 0.8 (1.3) | 1.7 (2.7) |
| Reported family history of mental illness | 36.4% | 80.9% | 70.0% |
| Previous or current psychotherapy | 27.2% | 100% | 95.0% |
| Currently taking psychoactive medication | 0.0% | 76.2% | 80.0% |
| Antidepressant |  | 71.4% | 70.0% |
| Antipsychotic |  | 9.5% | 35.0% |
| Tranquilizer |  | 9.5% | 5.0% |
| Stimulant |  | 9.5% | 15.0% |
| Anticonvulsant |  |  | 5.0% |
| Antiparkinsonian |  | 9.5% |  |
| Sedative |  | 9.5% | 30.0% |
| Cannabinoid (prescribed) |  | 4.7% | 5.0% |
| Opioid antagonist |  |  | 5.0% |
| Opioid (prescribed) |  | 4.7% |  |
| Contraceptives | 36.4% | 33.3% | 35.0% |

*Supplementary Table 5. Diagnostic and Clinical Characteristics Across Recruitment Groups n* = 22 controls; 23 MDD; 18 MDD+BPD; HAMD = Hamilton Depression Rating Scale; MDD = major depressive disorder; PDD = persistent depressive disorder; BPD = borderline personality disorder; PTSD = posttraumatic stress disorder. Note, the MDD and MDD+ BPD group did not differ in depressive severity scores measured by the HAMD, *t* = .95, *p* = .60.
